# Immune activation following PD-L1 inhibitor plus chemoradiotherapy in locally advanced rectal cancer: a retrospective, single-arm study

**DOI:** 10.3389/fimmu.2025.1619043

**Published:** 2025-08-27

**Authors:** Shaoqing Fan, Zeming Zhao, Qingyu Meng, Haiqian Wang, Bin Yu, Wenbo Niu

**Affiliations:** ^1^ Department of General Surgery, The Fourth Hospital of Hebei Medical University, Shijiazhuang, Hebei, China; ^2^ Department of General Surgery, The Second Hospital of Hebei Medical University, Shijiazhuang, Hebei, China; ^3^ Department of Nursing, The Fourth Hospital of Hebei Medical University, Shijiazhuang, Hebei, China

**Keywords:** locally advanced rectal cancer, neoadjuvant chemoradiotherapy, tumor microenvironment, PD-L1 inhibitor, tumor-infiltrating lymphocytes

## Abstract

**Background:**

Locally advanced rectal cancer (LARC) is challenging due to high recurrence rates and poor responses to neoadjuvant chemoradiotherapy (nCRT). Combining nCRT with immunotherapy may enhance antitumor immunity by modifying the tumor microenvironment (TME). This study evaluates the efficacy of nCRT with PD-L1 inhibitor envafolimab in LARC and explores its impact on TME.

**Methods:**

In this retrospective, single-arm design study, 36 LARC patients (T3+/N1-2/M0) received long-course radiotherapy (50.4 Gy/28 fractions) with capecitabine, followed by two cycles of XELOX chemotherapy and envafolimab. Pathological complete response (pCR) and tumor regression grade (TRG) were assessed post-surgery. Immunohistochemical analysis quantified CD4+, CD8+ T cells, and CD56+ NK cell infiltration in paired pre- and post-treatment tumor tissues.

**Results:**

The pCR rate was 47.2% (17/36), with 94.4% and 86.1% achieving T- and N-downstaging. Post-treatment tumor-infiltrating lymphocytes (TILs) increased, with CD8+ T cells showing the most significant infiltration (Grade 3: +6 cases, P<0.05). Higher baseline TIL density correlated with better TRG outcomes (TRG0-2: 94.4% vs. TRG3: 5.6%).

**Conclusion:**

nCRT combined with envafolimab enhances immune cell infiltration, particularly CD8+ T cells, achieving high pCR rates in LARC. This approach enhances cytotoxic immunity while addressing immunosuppressive barriers. Further studies should explore strategies to overcome TME resistance.

## 1 Introduction

Colorectal cancer (CRC) is one of the most common malignant gastrointestinal tumors worldwide, with persistently high incidence and mortality rates. Epidemiological data indicate that the incidence of CRC is increasing at an annual rate of approximately 2%, and it ranks as the second leading cause of cancer-related deaths in developed countries (1). With socioeconomic development and the widespread adoption of a Westernized diet, a high-fat and high-protein diet has been significantly associated with the rising incidence of CRC (2). According to the latest data released by the National Cancer Center of China in 2022, CRC has become the second most prevalent malignancy in China, with mortality rates ranking fourth, making it a major public health concern (3).

Early diagnosis of CRC remains a major challenge, as the disease often presents with nonspecific or asymptomatic manifestations in its initial stages, such as abdominal pain, rectal bleeding, diarrhea, or iron deficiency anemia (4). Consequently, many patients are diagnosed at an advanced stage, significantly impacting treatment outcomes and prognosis (1). Colonoscopy is currently the most effective screening tool for early detection, offering high diagnostic accuracy. However, several limitations hinder its widespread clinical application: first, it requires specialized endoscopists for proper execution; second, the procedure is time-consuming; and third, it can cause patient discomfort and carries potential risks of complications (5). These factors collectively contribute to poor adherence to screening programs, limiting the effectiveness of early CRC detection and intervention.

LARC, typically defined as stage II–III disease (cT3–T4 and/or cN+ without distant metastasis, M0), is characterized by tumor invasion beyond the muscularis propria and regional lymph node involvement, which confer a high risk of local recurrence and distant metastasis. Accurate clinical staging is essential for guiding treatment decisions and is primarily performed using contrast-enhanced chest and abdominal computed tomography (CT) to exclude distant metastases, combined with high-resolution pelvic magnetic resonance imaging (MRI) to evaluate tumor invasion depth and nodal status (6). Radical surgery has long been the cornerstone of treatment for both early and locally advanced rectal cancer, aiming to achieve long-term survival through complete tumor resection. However, patients with LARC continue to face a significant risk of postoperative local recurrence (7). In recent years, the development of a multidisciplinary treatment (MDT) approach has led to the widespread adoption of nCRT, which has been shown to significantly reduce local recurrence rates and improve sphincter-preserving outcomes (8). Studies have demonstrated that nCRT not only facilitates tumor downstaging but also effectively controls micrometastases, thereby improving the feasibility and success of surgical resection. Clinical data indicate that approximately 20%–40% of patients achieve clinical complete response (cCR) following nCRT, with 0%–30% attaining pCR (8). For patients achieving cCR, nonoperative management strategies, such as the “watch-and-wait” approach, are being actively explored to avoid surgery-related complications and improve quality of life (9). However, precise patient selection for nCRT and optimization of treatment regimens remain key areas of ongoing research.

Despite the established benefits of nCRT, approximately 20-30% of patients with LARC exhibit poor responses, including tumor progression or metastasis during treatment (10). Furthermore, long-term survival benefits remain limited even among responders across all patient cohorts (11), underscoring the necessity for novel therapeutic strategies. Immunotherapy, recognized as a transformative approach in oncology, has consequently garnered increasing interest for integration with nCRT in LARC research. Emerging evidence supports the potential of this combination: a meta-analysis of neoadjuvant immunotherapy for non-metastatic colorectal cancer demonstrated that combining immunotherapy with nCRT significantly improves pCR and major pathological response (MPR) rates (12). Consistent findings were reported by Xiao et al. in their cohort of MMR (mismatch repair)-deficient patients (13). Moreover, Xiao et al. also reported that in patients with mismatch repair-proficient/microsatellite stable (pMMR/MSS) LARC—a subgroup typically refractory to single-agent immunotherapy—the combination of long-course chemoradiotherapy (LC-CRT) and the PD-1 inhibitor sintilimab achieved a pCR rate of 44.8% (13). This substantially exceeds historical pCR rates achieved with nCRT alone (8). These findings highlight the clinical potential of immunotherapy-based combinations to overcome treatment resistance and improve outcomes in LARC.

The TME is increasingly recognized as a critical mediator of therapeutic response. Modulation of the TME is believed to be a key mechanism underlying the efficacy of combined nCRT and immunotherapy. Evidence indicates that nCRT remodels the TME by altering immune cell infiltration; for example, studies demonstrate increased abundance of CD4+ T cells, CD8+ T cells, and CD56+ natural killer (NK) cells within LARC tumors following nCRT, suggesting immunomodulatory effects (14). Immunotherapy, particularly PD-1/PD-L1 blockade, may potentiate these effects by reversing immune suppression within the TME, thereby enhancing the cytotoxic activity of TILs (15). Consequently, the synergy between nCRT and immunotherapy in modulating the TME to enhance antitumor immunity provides a strong rationale for further investigation. TILs, particularly CD8^+^ T cells, are key effectors of antitumor immunity. CD8^+^ T cells directly recognize and kill tumor cells via perforin and granzyme release, while their infiltration density correlates with better tumor regression and prognosis (16, 17). However, in many LARC, especially pMMR/MSS subtypes, TIL infiltration is insufficient, and the TME is immunosuppressive—limiting the efficacy of single-agent immunotherapy (18).

nCRT can remodel the TME by inducing immunogenic cell death, releasing tumor antigens, and promoting T cell recruitment (19). Combining nCRT with PD-L1 inhibitors (e.g., envafolimab) may synergistically enhance CD8^+^ T cell infiltration and activity: nCRT increases antigen presentation and PD-L1 expression on tumor cells, while PD-L1 blockade reverses T cell exhaustion (20). This study thus explores whether this combination improves pCR rates by boosting CD4^+^, CD8^+^ T cells, and CD56^+^ NK cell infiltration in LARC”.

Based on these findings, this study aims to assess neoadjuvant immunotherapy combined with chemoradiotherapy in LARC patients and its impact on the TME. We included patients who underwent neoadjuvant immunotherapy and assessed clinical outcomes by comparing tumor staging before and after treatment, tumor regression rate, and pCR rate. Additionally, we utilized immunohistochemical staining to analyze the dynamic changes in TILs before and after nCRT, thereby elucidating the immune regulatory mechanisms of the TME during treatment. Our ultimate goal is to identify immune biomarkers predictive of nCRT response in LARC patients and provide a theoretical foundation for the clinical application of combined neoadjuvant chemoradiotherapy and immunotherapy.

## 2 Materials and methods

### 2.1 Study population

The sample size of the present study was determined based on 36 eligible patients who underwent rigorous screening against predefined inclusion and exclusion criteria at the Fourth Hospital of Hebei Medical University between January 1, 2022, and December 31, 2024. These 36 patients constituted all cases meeting the full set of screening criteria during the specified period. Owing to the retrospective and exploratory nature of this analysis, coupled with the paucity of prior data on the specific efficacy of envafolimab in this patient cohort, no statistical assumptions or power calculations were employed during the study planning phase. Complete clinical and pathological data were retrieved from the hospital’s medical records and pathology department.

Histopathological evaluation was conducted by two experienced pathologists who independently reviewed hematoxylin and eosin (HE)-stained sections. Representative formalin-fixed paraffin-embedded (FFPE) blocks were selected for immunohistochemical (IHC) staining of CD4+, CD8+ T, and CD56+ NK cells. Clinical and pathological parameters included sex, age, tumor location, preoperative staging, postoperative staging, and TRG.

This study was approved by the Ethics Committee of the Fourth Hospital of Hebei Medical University, and informed consent was obtained from all patients.

### 2.2 IHC

IHC staining was performed on representative formalin-fixed, paraffin-embedded tissue sections (4 μm thick) using the Benchmark ULTRA automated staining system (Ventana Medical Systems, USA) according to the manufacturer’s standardized protocol. Heat-induced epitope retrieval was conducted using a citrate-based buffer (pH 6.0) as a preprocessing step. Endogenous peroxidase activity was blocked using 3% hydrogen peroxide. Sections were incubated with primary antibodies at room temperature, followed by immunodetection using the UltraView DAB detection kit (Ventana Medical Systems), which employs a multimer-based HRP system with DAB as the chromogen. Hematoxylin was used for counterstaining.

The primary antibodies used are summarized in **Table 1**. Appropriate positive and negative controls were included in each staining batch to ensure specificity and reliability of immunostaining.

**Table 1:** Primary antibodies used in the study.

Antibody	Source	Clone	Dilution
CD4+	Thermo Fisher Scientific (USA)	UMAB64	1:100
CD8+	Leinco Technologies (USA)	MX117	1:100
CD56+	ZSGB-BIO (China)	UMAB83	1:100

TILs were assessed under high-power fields (×400 magnification) and quantified as the average number of cells per mm² across five randomly selected fields. Based on TIL density, infiltration levels were classified into three grades:

- Low infiltration (Grade 1): < 50 lymphocytes/mm²
- Moderate infiltration (Grade 2): 50–100 lymphocytes/mm²
- High infiltration (Grade 3): > 100 lymphocytes/mm²

### 2.3 Inclusion criteria

Patients were included in the study if they met the following criteria:

1. Age between 18 and 75 years;
2. Histopathologically confirmed diagnosis of rectal cancer;
3. Radiological staging of T3+ and/or N1–2 with M0 disease;
4. Received neoadjuvant chemoradiotherapy combined with envafolimab followed by radical surgery.
5. MSI (Microsatellite instability) status was not used as an inclusion criterion. Patients were enrolled regardless of MSI proficiency, as this study aimed to explore the general efficacy of the combined regimen in unselected LARC.

### 2.4 Exclusion criteria

Patients were excluded if they met any of the following conditions:

1. Prior treatment for rectal cancer (e.g., surgery, chemotherapy, radiotherapy, or immunotherapy) before the neoadjuvant chemoradiotherapy;
2. Presence of multiple concurrent malignancies.

### 2.5 Statistical analysis

Statistical analyses were performed using SPSS 20.0 software. The chi-square (χ²) test was used to compare categorical variables between groups. A p-value < 0.05 was considered statistically significant.

## 3 Results

### 3.1 Clinical and pathological characteristics

A total of 36 patients were included in this study, comprising 21 males and 15 females, with a median age of 48 years (range: 30–71 years). Among them, 19 patients had mid-to-upper rectal cancer (tumor located >5 cm from the anal verge), while 17 had low rectal cancer (tumor located <5 cm from the anal verge).

Preoperative clinical staging was assessed using contrast-enhanced rectal MRI and contrast-enhanced chest and abdominal CT. Among the included cases, 11 patients were classified as stage II and 25 as stage III. MSI status and mismatch repair (MMR) proficiency (pMMR/dMMR) were not assessed in this study due to limited pathological resources, which limits subgroup analyses based on these biomarkers. All patients underwent long-course preoperative radiotherapy (50.4 Gy in 28 fractions) with concurrent oral capecitabine (825 mg/m², twice daily). Following radiotherapy and prior to surgery, patients received two cycles of XELOX chemotherapy combined with envafolimab (a PD-L1 inhibitor).

The detailed clinical and pathological characteristics of the patients are summarized in **Table 2**.

**Table 2:** Clinical characteristics and pathological types.

Clinical Characteristics	N (%)
Gender	
Male	21 (58.3%)
Female	15 (41.7%)
Age	
<50 years	19 (52.8%)
≥50 years	17 (47.2%)
Tumor Distance from Anal Verge	
≤5 cm	21 (58.3%)
>5 cm	15 (41.7%)
T Stage	
cT2	1 (2.8%)
cT3	25 (69.4%)
cT4	10 (27.8%)
N Stage	
cN0	11 (30.6%)
cN1	18 (50.0%)
cN2	7 (19.4%)

### 3.2 Efficacy evaluation

After preoperative neoadjuvant immunotherapy, the tumor downstaging rate for T staging was 94.4%, and for N staging, it was 86.1% (**Table 3**). Postoperative pathological specimens showed TRG as follows: TRG 0 was observed in 17 cases (47.2%), TRG 1 in 11 cases (30.6%), TRG 2 in 6 cases (16.7%), and TRG 3 in 2 cases (5.6%) (**Table 4**).

**Table 3:** Pathological characteristics before and after radiotherapy and postoperative pathology.

Pathological Characteristics	Pre-radiotherapy	Post-radiotherapy	Postoperative Pathology
T Stage			
T0	0	5	17
T1	0	11	12
T2	1	10	5
T3	25	7	1
T4	10	3	1
N Stage			
N0	11	27	26
N1	18	8	9
N2	7	1	1

**Table 4:** TRG). which was evaluated according to the AJCC/CAP 4-tier system.

Clinical Characteristics	N (%)
TRG	
TRG 0	17 (47.2%)
TRG 1	11 (30.6%)
TRG 2	6 (16.7%)
TRG 3	2 (5.5%)

TRG 0 indicating complete response with no residual tumor cells; TRG 1, near complete response with only rare residual tumor cells; TRG 2, partial response with residual tumor and evidence of regression; and TRG 3, poor or no response with extensive residual tumor.

### 3.3 Comparison of TIL grading of CD4+ T, CD8+ T, and CD56+ NK cells in rectal cancer tissue before and after neoadjuvant combination therapy

After therapy, the number of patients with low CD4^+^ TIL infiltration (Grade 1) decreased by 10, while moderate (Grade 2) and high (Grade 3) infiltration increased by 5 cases each. The change in CD4^+^ TIL distribution before and after therapy was statistically significant (P < 0.05).

After therapy, the number of patients with low CD8^+^ TIL infiltration (Grade 1) decreased by 12, while moderate (Grade 2) infiltration increased by 5 cases and high (Grade 3) infiltration increased by 7 cases. The change in CD8^+^ TIL distribution before and after therapy was also statistically significant (P < 0.05).

**Figure 1:**
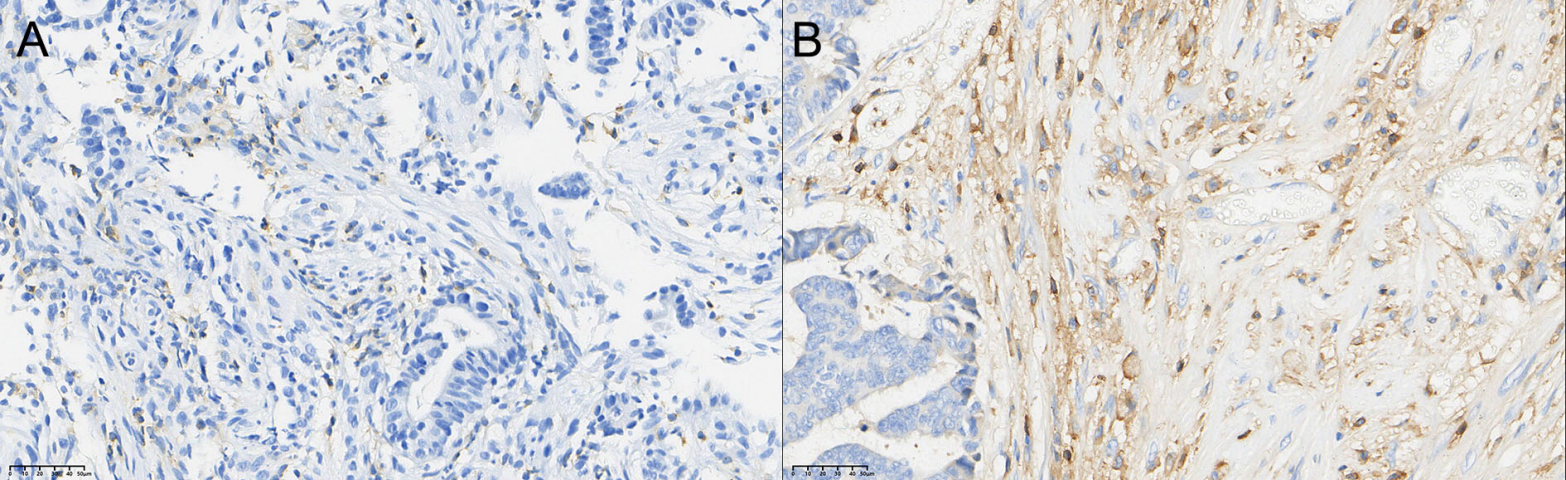
Comparison of the distribution of CD4+ T cells in rectal cancer tissues before **(A)** and after **(B)** neoadjuvant combination therapy (40×).

**Figure 2:**
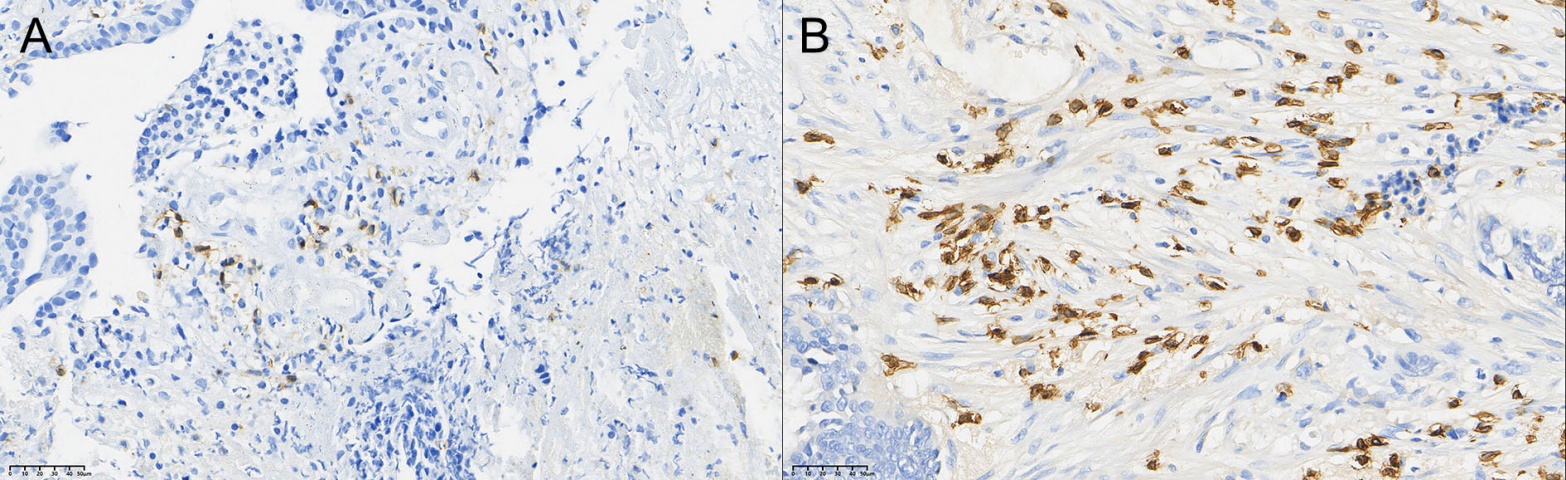
Comparison of the distribution of CD8+ T cells in rectal cancer tissues before **(A)** and after **(B)** neoadjuvant combination therapy (40×).

**Figure 3:**
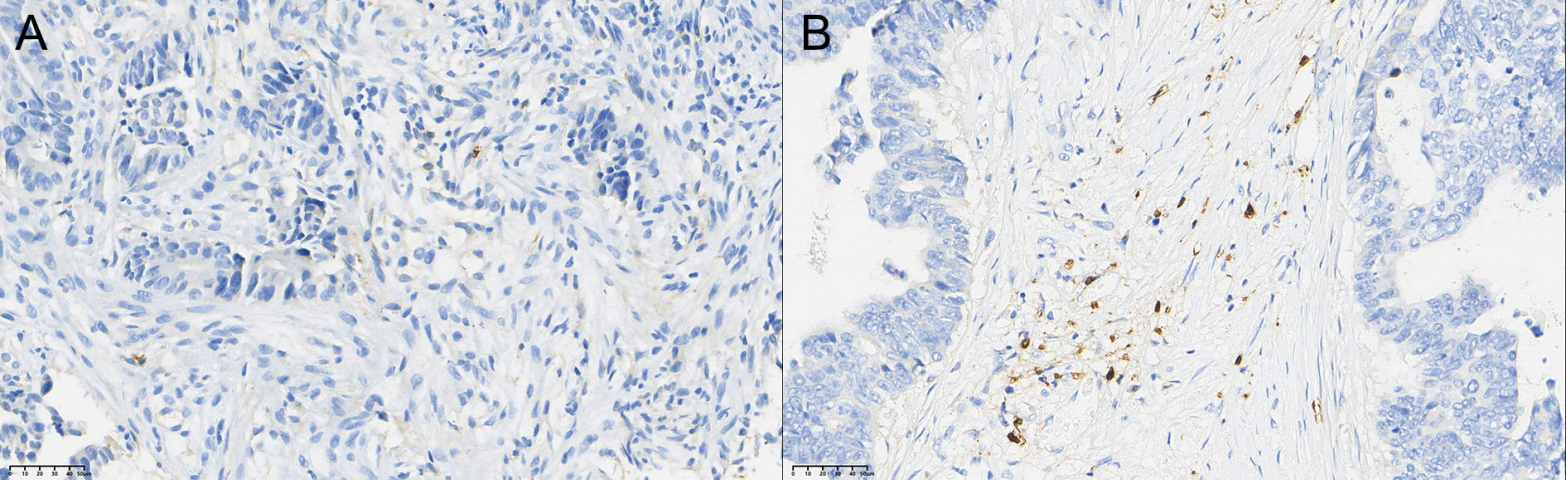
Comparison of the distribution of CD56+ NK cells in rectal cancer tissues before **(A)** and after **(B)** neoadjuvant combination therapy (40×).

After therapy, the number of patients with low CD56^+^ NK cell infiltration (Grade 1) decreased by 11, those with moderate infiltration (Grade 2) decreased by 5, and those with high infiltration (Grade 3) increased by 6. The change in CD56^+^ NK cell distribution before and after therapy was statistically significant (P < 0.05). Detailed data are presented in **Table 5** and **Figures 1**–**3**.

**Table 5:** Comparison of TIL Grading of CD4+ T, CD8+ T, and CD56+ Cells in Rectal Cancer Tissue Before and After Neoadjuvant combination therapy.

TIL Grading	1	2	3	χ²	P
CD4+ T					
Pre-radiotherapy	15	12	9	7.95	0.019
Post-radiotherapy	5	17	14		
CD8+ T					
Pre-radiotherapy	15	10	11	10.69	0.005
Post-radiotherapy	3	15	18		
CD56+					
Pre-radiotherapy	18	10	8	7.88	0.019
Post-radiotherapy	7	15	14		

### 3.4 Relationship between pre-radiotherapy CD4+ T, CD8+ T, and CD56+ TIL grading and local efficacy in rectal cancer

In rectal cancer tissues before radiotherapy, higher densities of CD4+ TIL, CD8+ TIL, and CD56+ TIL were associated with a greater proportion of patients achieving TRG0, TRG1, and TRG2 grades, while fewer patients exhibited TRG3. However, the differences were not statistically significant (P > 0.05), as shown in **Table 6**.

**Table 6:** Comparison of pre-radiotherapy CD4+ T, CD8+ T, and CD56+ TIL grading with local efficacy in rectal cancer.

TIL Grading	1	2	3	χ²	P
CD4+ T					
TRG 0,1,2	14	11	9	0.74	0.692
TRG 3	1	1	0		
CD8+ T					
TRG 0,1,2	13	10	11	3.99	0.135
TRG 3	2	0	0		
CD56+					
TRG 0,1,2	16	10	8	2.12	0.347
TRG 3	2	0	0		

## 4 Discussion

LARC remains a therapeutic challenge, with nCRT as the standard of care, yet 20-30% of patients exhibit poor responses, and long-term survival benefits remain limited (21). This underscores an urgent need to optimize treatment strategies by leveraging synergies between conventional therapies and immunotherapy. Our study addresses this gap by evaluating the efficacy of nCRT combined with the PD-L1 inhibitor envafolimab, with a focus on its impact on the TME and antitumor immunity—this represents the core innovation of our work. Our key finding is a high Pcr rate of 47.2% (17/36) in LARC patients treated with nCRT plus envafolimab, accompanied by significant T- (94.4%) and N-downstaging (86.1%). This pCR rate exceeds historical rates of 0-30% with nCRT alone (8) and compares favorably to similar combination strategies, such as Xiao et al.’s report of 44.8% pCR with sintilimab plus long-course chemoradiotherapy in pMMR/MSS LARC (13). The superior efficacy observed here likely stems from the synergistic modulation of the TME by nCRT and PD-L1 inhibition. Preoperative staging of rectal cancer is a critical factor in determining the treatment plan, primarily relying on imaging modalities such as CT, MRI, and endorectal ultrasound (ERUS). Currently, MRI is recommended internationally as the preferred method for preoperative staging of rectal cancer due to its high accuracy in assessing tumor invasion depth (T-stage) and lymph node metastasis (N-stage) (6). In this study, all cases underwent preoperative staging with rectal MRI, which was evaluated twice—once before and once 8 weeks after neoadjuvant immunotherapy. The results showed a significant downstaging of T-stage (94.4%) and N-stage (86.1%), highlighting the potent downstaging effect achieved by the combination of nCRT and PD-L1 inhibitor in locally advanced rectal cancer, which is consistent with the findings of Chalabi et al. (13). This suggests that the addition of PD-L1 inhibitor to standard nCRT may significantly enhance T and N downstaging compared to nCRT alone. The study also suggests that immunotherapy enhances the antitumor effect of chemoradiotherapy by activating immune responses in the tumor microenvironment.

However, despite the important role of MRI in rectal cancer staging, its accuracy in evaluating pCR following neoadjuvant chemoradiotherapy combined with a PD-L1 inhibitor remains limited. This study found that MRI had an accuracy of only 29.4% in assessing T0-stage, with many patients initially classified as T3 or T4 on MRI, later showing a lower pathological stage post-surgery. In contrast, this study shows that MRI’s assessment of N-stage is more accurate, with an accuracy of 94.4%, as its high soft tissue resolution allows clear visualization of lymph node morphological characteristics (22). In the future, combining functional imaging techniques (such as dynamic contrast-enhanced MRI and radiomics) with artificial intelligence algorithms may further enhance the accuracy of MRI in rectal cancer staging, particularly in evaluating the degree of tumor regression following neoadjuvant therapy that includes a PD-L1 inhibitor (23).

The degree of immune cell infiltration in rectal cancertissues plays a critical role in treatment response and prognosis. nCRT contributes to antitumor immunity not only by directly killing tumor cells but also by modulating the immune response within the TME. Studies have shown that nCRT-induced tumor cell necrosis and apoptosis release a significant amount of tumor antigens, which are captured and presented to T cells by antigen-presenting cells (such as dendritic cells), subsequently activating CD4+ and CD8+ T cells and triggering an immune response (24). In our study, patients treated with nCRT combined with a PD-L1 inhibitor showed a marked increase in CD4^+^ and CD8^+^ T cell infiltration following therapy. This enhancement is likely associated with antigen release induced by chemoradiotherapy and further amplified by immune checkpoint blockade, which relieves T cell exhaustion and facilitates sustained immune activation. Moreover, nCRT may contribute to T cell recruitment by inducing the release of proinflammatory cytokines such as IFN-γ and TNF-α (25), creating an immunostimulatory environment that supports the efficacy of PD-L1 inhibition.

CD4+ T cells primarily play an immunoregulatory role in antitumor immune responses. By secreting cytokines such as IL-2 and IFN-γ, they activate CD8+ T cells and B cells, thereby enhancing antitumor activity (26). Furthermore, CD4+ T cells can directly interact with antigen-presenting cells to promote the presentation of tumor antigens and the formation of immune memory (27). In our study, the increase in CD4^+^ T cells observed after combined nCRT and PD-L1 inhibitor treatment may contribute to enhanced antitumor immunity via immune surveillance and modulation of the tumor microenvironment. For example, CD4+ T cells can recognize tumor-specific antigens and indirectly kill tumor cells by activating other immune cells, such as macrophages and natural killer (NK) cells (28). At the same time, CD4+ T cells can regulate the tumor microenvironment by secreting cytokines like IL-12 and IL-21, further promoting CD8+ T cell infiltration and function (29).

CD8+ T cells are the primary effector cells in the antitumor immune response, capable of directly recognizing and killing tumor cells. After nCRT combined with a PD-L1 inhibitor, the increase in CD8+ T cells may play a role through mechanisms such as direct cytotoxicity, immune memory formation, and the chemotaxis of inflammatory cytokines. CD8+ T cells induce tumor cell apoptosis by releasing perforin and granzyme (30). Moreover, after eliminating tumor cells, CD8+ T cells can form memory T cells that provide long-term immunosurveillance for tumor recurrence (31). Inflammatory cytokines (such as CXCL9 and CXCL10) induced by nCRT can also attract CD8+ T cells to the tumor site, enhancing their cytotoxic effect (32). CD4+ and CD8+ T cells exert a synergistic effect in the antitumor immune response. CD4+ T cells support CD8+ T cell activation and function by providing co-stimulatory signals and cytokines (33). Additionally, CD4+ T cells can further promote the antitumor activity of CD8+ T cells by modulating immunosuppressive cells in the tumor microenvironment, such as Tregs and MDSCs (Myeloid-Derived Suppressor Cells) (34). This synergistic effect is particularly pronounced after nCRT, further enhancing the antitumor immune response.

CD56+ natural killer (NK) cells also play a crucial role in antitumor immunity. NK cells directly kill tumor cells by releasing cytotoxic granules (such as perforin and granzyme) and secreting cytokines like IFN-γ (35). Recent studies have shown that stress and damage induced by nCRT combined with a PD-L1 inhibitor in tumor cells can upregulate stress ligands (such as MICA/B) on the surface of tumor cells, thereby enhancing NK cell recognition and killing ability (36). Furthermore, CD4+ T cells can promote NK cell activation and proliferation by secreting cytokines like IL-2 and IL-15 (37). There is also a synergistic effect between CD8+ T cells and NK cells. CD8+ T cells enhance NK cell cytotoxicity by secreting IFN-γ, while NK cells remove immunosuppressive cells, such as Tregs and MDSCs, creating a more favorable microenvironment for CD8+ T cells (38). CD4+, CD8+ T cells, and CD56+ NK cells collaborate in the antitumor immune response. CD4+ T cells enhance the activation and function of CD8+ T cells and NK cells by providing co-stimulatory signals and cytokines (39). This synergistic effect is particularly significant after combined nCRT and PD-L1 inhibitor treatment, further enhancing the effectiveness of the antitumor immune response. The application of immunotherapy in CRC is often limited by the TME, especially in most pMMR/MSS tumors. These tumors typically exhibit insufficient lymphocytic infiltration and an immunosuppressive microenvironment, leading to poor responses to immune checkpoint inhibitors, such as PD-1/PD-L1 blockers (40). However, recent studies have shown that radiotherapy and chemotherapy can modulate the TME through various mechanisms, enhancing the efficacy of immunotherapy (41). Radiotherapy induces immunogenic cell death in tumor cells, releasing tumor antigens and damage-associated molecular patterns (DAMPs), which activate antigen-presenting cells (such as dendritic cells) and promote the infiltration and activation of antigen-specific CD8+ T cells (24). Additionally, radiotherapy can upregulate PD-L1 expression on tumor and immune cells, making tumors that were previously resistant to PD-1/PD-L1 blockade sensitive to therapy (42). In our study, combined nCRT and PD-L1 inhibitor therapy significantly increased the infiltration of CD4+, CD8+ T cells, and CD56+ NK cells in the TME, which is closely associated with radiotherapy-induced immune enhancement. Chemotherapeutic agents, such as 5-fluorouracil and oxaliplatin, not only exert direct cytotoxic effects on tumor cells but also enhance the antitumor immune response by upregulating PD-L1 expression on dendritic cells and promoting immune cell infiltration (43). Furthermore, chemotherapy can reduce the number of immunosuppressive cells, such as Treg cells and MDSCs, further improving the TME (44). These synergistic mechanisms suggest that the combination of chemotherapy and immunotherapy holds potential therapeutic value in pMMR/MSS rectal cancer. In a study by Xiao et al., pMMR LARC patients treated with long-course chemoradiotherapy (LC-CRT) combined with sintilimab achieved a pCR rate of 44.8% (13). This result indicates that the combination of chemoradiotherapy and immunotherapy significantly enhances the immune response in pMMR/MSS tumors. In our study, the pCR rate in pMMR patients was further increased to 47.2%, likely attributed to the tumor microenvironment remodeling and increased immune cell infiltration induced by nCRT.

In studies on rectal cancer before nCRT combined with PD-L1 inhibitor therapy, it has been observed that higher densities of CD4+ TILs, CD8+ TILs, and CD56+ TILs in tumor tissues correlate with an increased proportion of patients achieving TRG0, 1, or 2 (complete or partial tumor regression) and a relative decrease in the number of TRG3 (no regression). This phenomenon suggests a positive correlation between the density of TILs and the tumor regression rate, which may be closely related to the effectiveness of antigen presentation and immune response in the TME. High-density CD4+ TILs, CD8+ TILs, and CD56+ NK cells are generally associated with stronger antitumor immune responses. CD8+ T cells directly kill tumor cells by recognizing tumor antigens, while CD4+ T cells enhance the function of CD8+ T cells and NK cells by providing co-stimulatory signals and cytokines (45). NK cells directly kill tumor cells by releasing cytotoxic granules, such as perforin and granzyme, and further activate T cells by secreting cytokines like IFN-γ (36). Therefore, high-density TILs may enhance the killing efficiency of tumor cells through synergistic effects, thereby improving the regression rate. Furthermore, antigen-presenting cells (APCs), such as dendritic cells and macrophages, present tumor antigens to CD8+ and CD4+ T cells through MHC I and MHC II molecules, initiating and sustaining the antitumor immune response (46). Radiotherapy may induce immunogenic cell death (ICD), releasing tumor antigens and DAMPs, which promote the maturation and antigen presentation of APCs (24). Therefore, high-density TILs may reflect more efficient antigen presentation and T cell activation processes, thus correlating with a higher tumor regression rate.

Although high-density TILs are typically associated with better prognosis, immunosuppressive factors in the TME (such as Treg cells, MDSCs, and immune checkpoint molecules) may limit their function. For example, Treg cells inhibit CD8+ T cell activity by secreting TGF-β and IL-10, while PD-L1 expression may mediate T cell exhaustion (34). In this study, although high-density TILs were associated with a higher proportion of TRG0-2, the presence of an immunosuppressive microenvironment may have diminished the statistical significance of this association.

In our study, nCRT combined with PD-L1 inhibitor therapy significantly enhanced the infiltration of immune cells, particularly CD8+ T cells and CD4+ T cells, into the TME, thus improving the therapeutic response in rectal cancer patients. Although nCRT combined with PD-L1 inhibitor therapy effectively promotes immune cell infiltration, we also observed that the presence of an immunosuppressive microenvironment might limit the functionality of immune cells, particularly through the actions of Treg cells, MDSCs, and immune checkpoint molecules within the tumor. Notably, while high densities of CD4+ T cells, CD8+ T cells, and CD56+ NK cells are positively correlated with higher tumor regression rates, the interplay of immunosuppressive factors may partially diminish the statistical significance of this effect. A key limitation is the lack of MSI/MMR status assessment. Previous studies have shown that dMMR tumors exhibit higher responsiveness to immunotherapy (47), while pMMR/MSS tumors often require combination strategies (13). Without this data, we cannot clarify whether the observed pCR rate (47.2%) is influenced by MMR status.

Future research must systematically integrate MSI/MMR status assessment to clarify subtype-specific influences on the observed pCR rate (47.2%) and divergent immune microenvironments. Subsequent efforts should prioritize overcoming subtype-defined immunosuppressive mechanisms, particularly identifying and targeting T-cell inhibitory pathways in pMMR/MSS tumors. Crucially, clinical trials evaluating PD-1/PD-L1 inhibitors combined with chemoradiotherapy must be stratified by MSI/MMR status. This is essential to define synergistic efficacy across subgroups and optimize combination strategies, especially for the less responsive pMMR/MSS cohort. Integrating advanced functional imaging (e.g., DCE-MRI, radiomics) with molecular profiling offers a promising avenue for non-invasively monitoring dynamic treatment responses and immune activation across subtypes. Collectively, these approaches will advance personalized therapeutic strategies in rectal cancer.

## 5 Conclusion

This study validates the positive impact of nCRT combined with immunotherapy on the immune microenvironment of rectal cancer, particularly through increased infiltration of CD4+, CD8+ T cells, and CD56+ NK cells, which enhances the antitumor immune response. However, the presence of an immunosuppressive microenvironment may affect immune cell function and therapeutic response. Future research should focus on reversing immunosuppressive mechanisms, the combined use of immune checkpoint inhibitors, and further advancements in imaging technologies to provide more effective treatment options for rectal cancer patients, particularly in pMMR/MSS tumors.

## Data Availability

The raw data supporting the conclusions of this article will be made available by the authors, without undue reservation.
